# Compareads: comparing huge metagenomic experiments

**DOI:** 10.1186/1471-2105-13-S19-S10

**Published:** 2012-12-19

**Authors:** Nicolas Maillet, Claire Lemaitre, Rayan Chikhi, Dominique Lavenier, Pierre Peterlongo

**Affiliations:** 1 INRIA Rennes - Bretagne Atlantique/IRISA, EPI GenScale, Rennes, France; 2 ENS Cachan/IRISA, EPI GenScale, Rennes, France

## Abstract

**Background:**

Nowadays, metagenomic sample analyses are mainly achieved by comparing them with *a priori *knowledge stored in data banks. While powerful, such approaches do not allow to exploit unknown and/or "unculturable" species, for instance estimated at 99% for Bacteria.

**Methods:**

This work introduces Compareads, a *de novo *comparative metagenomic approach that returns the reads that are similar between two possibly metagenomic datasets generated by High Throughput Sequencers. One originality of this work consists in its ability to deal with huge datasets. The second main contribution presented in this paper is the design of a probabilistic data structure based on Bloom filters enabling to index millions of reads with a limited memory footprint and a controlled error rate.

**Results:**

We show that Compareads enables to retrieve biological information while being able to scale to huge datasets. Its time and memory features make Compareads usable on read sets each composed of more than 100 million Illumina reads in a few hours and consuming 4 GB of memory, and thus usable on today's personal computers.

**Conclusion:**

Using a new data structure, Compareads is a practical solution for comparing *de novo *huge metagenomic samples. Compareads is released under the CeCILL license and can be freely downloaded from http://alcovna.genouest.org/compareads/.

## Introduction

The past five years have seen the arrival of High Throughput Sequencing (HTS), also known as Next-Generation Sequencing (NGS). These technologies drastically lowered sequencing costs and increased sequencing throughput. They radically changed molecular biology and computational biology, as data generation is no longer a bottleneck. In fact, nowadays a major challenge is the analysis and interpretation of sequencing data [1]. HTS democratized access to sequencing to almost all biological labs over the world. It also opened the doors to new techniques such as ChipSeq [2], ClipSeq [3], RadSeq [4] and the topic of this work, metagenomics [5].

Metagenomics, also known as "environmental genomics", provides an alternative to traditional single- genome studies for exploring the microbial world. Most microorganisms (up to 99% of Bacteria [6]) are unknown and possibly "unculturable". Even if traditional genomics sequencing methods are well studied, they are not suited for environmental samples, because of the need to cultivate clones. By sequencing uncultured genomes directly from environmental samples, metagenomics offers new ways to study this unexplored diversity.

HTS technologies provide fragments of sequences (called reads) of length a few hundred base pairs without any information about the locus nor the orientation on the molecule they come from. In the metagenomic context, an additional difficulty comes from the fact that each read may belong to any species.

Nowadays, it is difficult to assemble complex metagenomes (such as soil or water metagenomes) into longer consensus sequences, because reads from different species may be merged into one chimeric sequence. Mende and colleagues [7] showed that for a 400-genomes metagenome, using simulated Illumina reads, 37% of the assembled sequences were chimeric. Thus currently, reads from metagenomes are used to estimate the biodiversity [8] or may be compared to known databases, providing information with respect to the current scientific knowledge [9,10]. Another way to exploit two or more metagenomic datasets is to compare them together, enabling to understand how genomic differences are related to environmental ones (biotopes localizations and/or time spent after an event).

Comparative metagenomics usually deals with many aspects, such as sequence composition, *i.e*. GC content [11], and genome size [12], taxonomic diversity [13], functional content [14], etc. Several methods are currently developed for comparative metagenomics analyses. Some are based on statistical methods with a large number of descriptive variables, *e.g*. principal component analysis (PCA).

To the best of our knowledge, there is no software designed to compare two or more metagenomic samples at the read level, *i.e*. to identify reads that are shared or similar between samples. This can be simply used to compute a similarity measure between samples such as the number or percentage of similar reads between pairs of samples. When dealing with more than two samples, this would enable among others to classify metagenomics samples based on their raw reads content. One could use the popular tool BLAST to align reads in an all-vs-all way, however it is not designed specifically to this task, and more importantly, it cannot cope in time and memory with the size of nowadays metagenomic samples obtained with current sequencing technologies. For instance, with the aim of exploring the diversity of small eukaryotes in the oceans all over the world, the expedition "Tara Ocean" [15] is generating more than 400 metagenomic samples containing each around 100 million short reads, that will need to be compared to each other.

Here, we introduce a time and memory-efficient method for extracting similar reads between two metagenomic datasets. The similarity is based on shared *k*-mers (words of length *k*). In order to fit with current memory capacities, the data structure we use is a modified version of a Bloom filter [16]. Bloom filters have recently been used in bioinformatics, notably for assembly graph partitioning [17], which enabled to perform metagenomic *de novo *assembly using 30x less memory.

This manuscript presents two main contributions: (**I**) a new algorithm, called Compareads, which computes the similarity measure between two metagenomics datasets; (**II**) a new simple but extremely efficient data structure based on the Bloom filter for storing the presence/absence of *k*-mers in huge datasets. The manuscript is organized as follows: in Section "*Methods*", we depict the Compareads algorithm and the new data structure. In Section "*Results*" we provide results both about the data structure and about Compareads, showing the efficiency of our approach in term of computation time, memory and biological accuracy.

## Methods

**Preliminaries and definitions **A *sequence *is composed by zero or more symbols from an alphabet ∑. In this work, as we are dealing with DNA, ∑ = {*A, C, G, T*}. A sequence *s *of length *n *on ∑ is denoted also by *s*[0]*s*[1] . . . *s*[*n *- 1], where *s*[*i*] ∈ ∑ for 0 ≤ *i < n*. We denote by *s*[*i*, *j*] the *substring s*[*i*]*s*[*i *+ 1] . . . *s*[*j*] of *s*. In this case, we say that the substring *s*[*i*, *j*] occurs at position *i *in *s*. We call *k-mer *a sequence of length *k*, and *s*[*i*, *i *+ *k *- 1] is a *k*-mer occurring at position *i *in *s*.

**Overview of **Compareads Compareads is designed for finding similar sequences between two read sets. This basic operation may appear extremely simple. However, it has to be highly efficient, in term of computation time and memory footprint, in order to scale with huge metagenomics datasets.

In order to perform efficiently this operation, Compareads indexes *k*-mers and uses a rough but efficient notion of "*similar sequences*" defined as follows:

**Definition 1 (shared *k*-mer) ***Two sequences s*_1 _*and s*_2 _*share a k-mer if and only if *∃(*i*_1_, *i*_2_) *such that s*_1_[*i*_1_, *i*_1 _+ *k *- 1] = *s*_2_[*i*_2_, *i*_2 _+ *k *- 1].

**Definition 2 (Similar sequences) ***Given integers k and t*, *two sequences s*_1 _*and s*_2 _*are said similar if and only if they share at least t non overlapping k-mers*.

In a few words, given two read sets *A *and *B*, the goal of the Compareads algorithm is to find the subset of reads from *A *which are similar to a read in *B *such set being denoted by $\left(A\vec{\cap }B\right)$ As it is a heuristic (see Section "*Dealing with false positives*"), our algorithm outputs an over-approximation of set $\left(A\vec{\cap }B\right)$ denoted by .

### Computing 

Compareads computes  in two steps. **The indexing **step consists in storing in memory all *k*-mers having at least one occurrence in the set *B*. The **query **step processes reads from set ***A ***one by one. For a read *r *∈ *A*, the index is used to test the presence in the set *B *of each *k*-mer of *r*. If at least *t *non-overlapping *k*-mers are returned as present, then the read *r *is inserted in . The main practical challenge faced by Compareads is to index the possibly huge volume of *k*-mers contained in *B*. The data structure must therefore fulfill three criteria: it must be quick to build, have a low memory footprint and be quick to request. Section "*The Bloom Data Structure index*" describes the chosen probabilistic data structure, based on a Bloom filter.

**Limiting the indexing space **To control the approximation error (see Section "*Dealing with false positives*"), the indexing phase is interrupted whenever the volume of *k*-mers in the first reads of *B *exceeds a fixed value *n*. The query phase is then performed on the whole *A *dataset. This phase returns a partial intersection between *A *and a first chunk of reads from *B*. The remaining partial intersections between *A *and the next chunks of reads from *B *(each representing a volume of *n k*-mers or less) are sequentially computed, until all the reads from *B *have been indexed. Eventually, Compareads returns the union of all partial intersections. Note that, in terms of results, this partitioning approach is strictly equivalent to performing a complete indexing of *B *then a query of all the reads from *A*. To avoid redundant computations, reads from *A *considered as "similar" in one of the partial intersections are tagged using a bitvector and are not queried further.

**Time complexity **Let *n_A _*and *n_B _*be the number of *k*-mers respectively in set *A *and set *B*. Computing  is done in time *O*(*n_B_*) (indexing) + $O\left(n_{A}\times \frac{n_{B}}{n}\right)$ (query). The $\frac{n_{B}}{n}$ term is due to the limitation of the indexing space.

### Ad hoc data structure

The index data structure we use is based on a Bloom filter, specially designed for the task of storing efficiently a huge set of *k*-mers, while being fast to build and to query. We shortly recall in Section "*Bloom filter*" what a Bloom filter is before describing, in Section "*The Bloom Data Structure index*", our data structure called BDS.

#### Bloom filter

A Bloom filter is a probabilistic data structure designed to test the membership of elements in a set [16]. It consists of an array of *m *bits, all initialized to zero, and a set of hash functions. Each hash function maps an element to a single position in the array. Each element is associated, through the values of the hash functions, to several positions in the array. To insert an element in the structure, the bits in the array associated to this element are all set to one. The structure answers membership queries by checking whether all the bits in the array associated to an element are set to one.

This data structure is probabilistic in nature, as false positives are possible. Even if an element is not in the set, its bits in the array may still be all set to one. This is because the bits associated to an element may independently be associated to other elements. Hence, the Bloom filter returns a wrong answer with non-zero probability. This probability is the *false positive rate*. An asymptotic approximation of the false positive rate is 0.6185*^m/n^*, assuming *n *elements are inserted in the *m*-bits array, and (ln 2 · (*m/n*)) hash functions are used [18]. False negatives never occur: if an element belongs to the set, the Bloom filter always answers positively. Bloom filters are space-efficient: only (*n *log_2 _*e *· log_2_(1*/ε*)) bits are required to support membership queries for *n *elements with a false positive rate of *ε *[18].

#### The Bloom Data Structure index

In this article, we consider a slightly different variation of Bloom filters: instead of using a single array of bits, each hash function corresponds to a distinct array, disjoint from all other functions. In terms of performance, with uniform hash functions, this variation is asymptotically equivalent to the original definition [18]. To avoid confusion with classical Bloom filters, we refer to this variation as BDS, standing for Bloom Data Structure.

**Particular hash functions **The hash functions used in this framework are a specific family of functions, which can be efficiently computed on consecutive *k*-mers. We consider the set of functions which map a *k*-mer to a bit sequence of length *k*, where each nucleotide is associated to a bit set to 0 or 1, depending only on its type (A, C, G or T). An exhaustive enumeration, in equations 1 and 2, shows that there exists only 7 functions in this set. We can distinguished two types, the first three, *f*_1_, *f*_2 _and *f*_3_, are said to be *balanced *(equation 1), whereas the other four are said to be *unbalanced *(equation 2)

(1) $$f_{j}:\Sigma^{k}\to {\left\{0,1\right\}}^{k}:\forall i\in \left[1,\;k\right]\left\{\begin{array}{c} \;f_{1}\left(s\right)\left[i\right]=0\;if\;s\left[i\right]=A\;\text{or}\;C\;f_{1}\left(s\right)\left[i\right]=1\;\text{otherwise}\\ \;f_{2}\left(s\right)\left[i\right]=0\;if\;s\left[i\right]=A\;\text{or}\;G\;f_{2}\left(s\right)\left[i\right]=1\;\text{otherwise}\\ \;f_{3}\left(s\right)\left[i\right]=0\;if\;s\left[i\right]=A\;\text{or}\;T\;f_{3}\left(s\right)\left[i\right]=1\;\text{otherwise} \end{array}\right)$$

(2) $$f_{j}:\Sigma^{k}\to {\left\{0,1\right\}}^{k}:\forall i\in \left[1,\;k\right]\left\{\begin{array}{c} \;f_{4}\left(s\right)\left[i\right]=0\;\text{if}\;s\left[i\right]=A\;f_{4}\left(s\right)\left[i\right]=1\;\text{otherwise}\\ \;f_{5}\left(s\right)\left[i\right]=0\;\text{if}\;s\left[i\right]=C\;f_{5}\left(s\right)\left[i\right]=1\;\text{otherwise}\\ \;f_{6}\left(s\right)\left[i\right]=0\;\text{if}\;s\left[i\right]=G\;f_{6}\left(s\right)\left[i\right]=1\;\text{otherwise}\\ \;f_{7}\left(s\right)\left[i\right]=0\;\text{if}\;s\left[i\right]=T\;f_{7}\left(s\right)\left[i\right]=1\;\text{otherwise} \end{array}\right)$$

One important property of these functions is that there is a simple relationship between the hash values of two consecutive *k*-mers in a read. One can see that the hash value of the next *k*-mer can be quickly computed, by left-shifting the binary sequence of the previous hash value and appending an extra bit. These functions are not classical hash functions, yet we show that they exhibit good hashing properties when applied to *k*-mers. In Section "*Practical performance of the *BDS, *comparison with other data structures*", the performance of these functions is compared with that of a classical hash function in terms of computation time, and false positive rate in the BDS.

### The Compareads pipeline

Computing  is asymmetrical. Indeed  does not contain the reads from *B *which are similar to reads in *A*. For doing this, one needs to compute also . In practice, for fully and symmetrically comparing two sets *A *and *B *we apply a pipeline slightly more complicated than simply  followed by . This whole pipeline, designed for reducing a heuristic effect is described in Section "*False positives due to k-mer shared between a read and a dataset*".

**Similarity measure **While comparing read sets *A *and *B*, the result provided by Compareads is composed of two sets:  and . Then, a similarity measure between the two datasets is computed as follows:

 where |*X*| denotes the cardinality of the set *X*.

### Dealing with false positives

Our approach may generate false positives for two reasons we describe in the two upcoming sections, which also expose solutions for limiting these effects.

#### False positives due to k-mer shared between a read and a dataset

Using *t >*1, Compareads algorithm can call similar sequences that do not respect strictly the definition of similarity given in definition 2. Indeed, steps described in Section "*Computing *" detect reads from *A *that share at least *t k*-mers with reads from *B*. This is less stringent than finding reads from *A *that share at least *t k*-mers with at least one read from set *B*. In fact, the *t k*-mers found in read *A *are possibly spread over two or more distinct reads from set *B*.

This issue can be mitigated by performing the following steps to compute both  and :

1. Compute , storing the results in a set denoted by .

2. Compute  storing the results in a set denoted by .

3. Compute  storing the results in a set denoted by .

In a few words, the two output datasets  and  are obtained by applying the fundamental operation  between a query and a read set being itself already the result of the asymmetrical  operation. This enables to remove some false positives due to *k*-mers spread over several reads.

**Figure 1:**
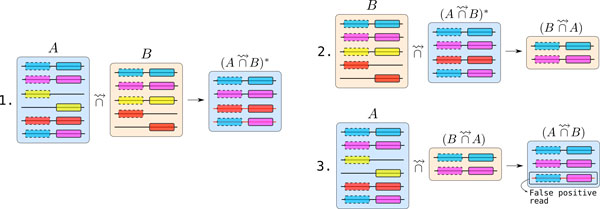
**The Compareads pipeline**. Representation of the three steps while comparing symmetrically read sets *A *and *B*. In each set, reads are represented by horizontal lines. On each read one or two shared *k*-mers are represented by rectangles.

The example presented in Figure 1 illustrates this issue for the case *t *= 2. The two first reads of sets *A *and *B *are similar. They are classically output by Compareads respectively in  and . The two next reads contain only one shared *k*-mer (yellow) with reads of set *B*, they are discarded. The next read of set *A *contains two (red) shared *k*-mers with two distinct reads in set *B*. After a first comparison,  contains this false positive read. However, in step 2, while computing , these two reads are not conserved in . Thus, during step 3, the two red *k*-mers are not present anymore in set  and thus are not present in . They are thus correctly absent from the final results . However, the last read from set *A *is a case of false positive. It contains *k*-mers spread over distinct reads from *B*, the latter belonging to . Thus, even during step 3, these two *k*-mers remain shared with reads from set  and are output in .

Note that in practice, the last set  is obtained by computing  instead of simply  (used here for simplifying the reading). This operation provides the same result but is computed faster as .

As outlined in the example Figure 1, this pipeline still yields some false positives. These are characterized by *t *shared *k*-mers with at least two distinct reads from the indexed dataset *B*, themselves considered as similar to reads of set *A*. Even if this side effect is difficult to assess, we show in Section "*Comparison with a classical approach using *BLAST" that Compareads provides trustworthy results, highly similar to a classical approach, on several real datasets.

#### Bloom filter false positives

As exposed in Section "*The Bloom Data Structure index*", the BDS index is a probabilistic data structure, that may consider a *k*-mer as indexed while this is not the case (*i.e*. a false positive or FP). Here, we analyse the variations of the false positive rate for each hash function and their combinations with respect to the parameter *k *and the number *n *of distinct indexed *k*-mers. This enables to determine optimal parameters and appropriate combination of functions, that give the best trade-off between memory usage and false positive rate.

**FP probablity for each function **Assuming the nucleotide composition of the indexed *k*-mers and of the query *k*-mers are unbiaised, we can easily compute the probability, *P_FP_*(*f_i_, k, n*), for any query *k*-mer to be a false positive with one of the seven hash functions, *f_i _*(see Additional file 1 for details). The expression of this probablity is presented in equation 3 for a balanced hash function, and 4 for an unbalanced one.

(3) $$\forall i\in \left\{1,2,3\right\}\;P_{FP}\left(f_{i},\;k,\;n\right)=1-{\left(1-\frac{1}{2^{k}}\right)}^{n}$$

(4) $$\forall i\in \left\{4,5,6,7\right\}\;P_{FP}\left(f_{i},\;k,\;n\right)=\overset{k}{\underset{x=0}{\sum }}\left(\begin{array}{c} k\\ x \end{array}\right)a_{x}\left(1-{\left(1-a_{x}\right)}^{n}\right)\;\;with\;a_{x}={\left(\frac{1}{4}\right)}^{x}{\left(\frac{3}{4}\right)}^{k-x}$$

**Figure 2:**
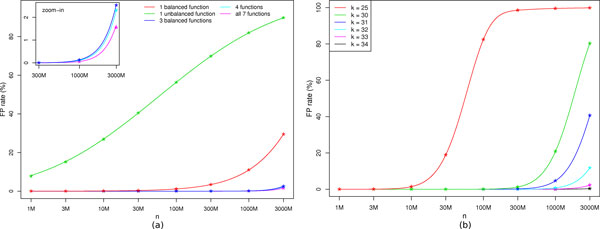
**BDS false positive rate w.r.t. hash functions (a) and *k*-value (b)**. FP rate as a function of the number of indexed *k*-mers (in log scale). Plain lines correspond to theoretical predictions, whereas star points correspond to empirical values obtained with simulations. **(a) **This figure was obtained for *k *= 33 for balanced and unbalanced functions and some combinations of them. The combination entitled "4 functions" is composed of the 3 balanced functions plus one unbalanced. **(b) **For several values of *k*, this figure was obtained for a combination of 4 hash functions: all three balanced plus one unbalanced.

We have plotted in Figure 2a the theoretical FP rate for both types of hash functions, and we can see that balanced functions give much less false positives than unbalanced ones. This is due to the fact that balanced functions distribute the hash codes uniformly over the 2*^k ^*bit-array, while this not the case for the unbalanced ones.

**FP probablity for a combination of functions **One important property of the balanced hash functions is that there do not exist two distinct *k*-mers that have the same couple of hash codes with any two of these functions. This implies that, in terms of false positives, two balanced functions have limited interferences with each other (see details in Additional file 1). The probability of FP can then be easily computed as follows:

(5) $$P_{FP}\left(f_{1}\cap f_{2}\cap f_{3},\;k,\;n\right)\;\underset{\sim }{<}\;{\left(1-{\left(1-\frac{1}{2^{k}}\right)}^{n}\right)}^{3}$$

This "independence" property implies also that combining these 3 functions in our BDS is a very efficient strategy to reduce the FP rate, as can be seen in Figure 2a, especially for large values of *n*

Concerning the unbalanced functions, such property does not hold, since it is possible to find couples of distinct *k*-mers that share the same couple of hash codes for at least 2 of the unbalanced functions, or for one balanced function and at least one unbalanced. Therefore the theoretical FP rate of unbalanced functions is much more difficult to compute. We performed simulations to compute an empirical FP rate. We found that empirical results are very close to the formula obtained by multiplying the individual probabilities, *i.e*. assuming complete independence between all functions (Figure 2a). For details about how empirical results were obtained, see Additional file 2.

**Choice of parameters **The comparison of these FP curves led us to choose the combination of the three balanced functions plus an unbalanced one. This choice is motivated by the fact that unbalanced functions are not essential, as they have a limited effect on the FP rate (Figure 2a). Since hash functions described in Section "*Particular hash functions*" have a fixed range, the memory used by the BDS depends only on the value of *k *and the number of hash functions used. Recall that each hash function is associated to a dedicated bit array which occupies 2*^k ^*bits. Using 7 hash functions, the BDS has a total memory footprint of 7 · 2*^k ^*bits and can be stored in 2*^k ^*bytes. When using only 4 hash functions, the BDS occupies 4 · 2*^k ^*bits and can be stored in twice less space (2^*k*-1 ^bytes).

For the chosen combination of functions, we plotted the FP rate as a function of *n *and for several values of *k *in Figure 2b. The larger is *k*, the less FP we get for a given number of indexed *k*-mers. Consequently, for large values of *k*, more *k*-mers can be indexed while maintaining a reasonable FP rate. However, the memory allocated to BDS grows with *k *and larger values of *k *increases the stringency of our similarity measure. We can see in Figure 2b, that using *k*-mers of size at least 30 enables to index at least 300 millions of *k*-mers with less than 2% of false positives.

For *k *= 33, when indexing up to one billion distinct *k*-mers, we obtain a theoretical upper bound of 0.13% of false positives (with 3 balanced functions, equation 5). The FP rate is even lower when adding one of the unbalanced function, we estimated it empirically to 0.114%. Thus, using 4 hash functions and *k *= 33 is a good set of parameters for indexing one billion distinct *k*-mers. With such parameters, the memory usage of Compareads is 4 GB.

## Results

### Practical performance of the BDS, comparison with other data structures

We propose here a comparative analysis of the BDS with other data structures. In the following, we show that classical non probabilistic data structures result in a worse time and memory performance, while in Section "*Comparison with other hash functions and with a classical Bloom filter*", we show that the BDS is the best suited for the problem of indexing huge amounts of *k*-mers.

#### Comparison with non probabilistic data structures: suffix array and hash table

Indexing *n *characters using the simplest version of a suffix array (not enhanced [19] and without LCP information) requires 5*n *bytes of memory [20]. Compared to our set of parameters where *n *= 1 billion, the memory footprint would be 5 × 10^9 ^bytes, *i.e*. 4.66 GB. While this is comparable to the BDS, the query time of the suffix array, *O*(*k *log *n*), is significantly worse.

An hash table can be used to store an exact set of *k*-mers. Such structure stores the *k*-mers explicitly, hence it requires at least $n\cdot \left\lceil \frac{2k}{8}\right\rceil \cdot 8$ bits (assuming no overhead), i.e. 16.5 GB for one billion of 33-mers. Thus, the BDS is four times more succinct.

#### Comparison with other hash functions and with a classical Bloom filter

**Time comparison with other hash functions **The hash functions defined for BDS were designed with speed in mind. In this paragraph, we compare them with a popular and fast hash function (Jenkins hash, specifically hashlittle2 from http://burtleburtle.net/bob/c/lookup3.c). We simulated 1 millon of 100-bp reads, where each nucleotide is drawn uniformly and independently. To simulate the behavior of computing hashes for the BDS, 4 hash values were computed for each 33-mer. For the hashlittle2 function, we simulated this behavior by computing 4 hashes with 4 different initial values. We recorded the time required to compute the hashes for all the 33-mers present in the reads, averaged over 3 executions. Computing the hash with the hashlittle2 function took 13.1 seconds (5.2 MHashes/s), whereas for the BDS hash functions, the same computation took 1.4 seconds (49.8 MHashes/s). Hence, the BDS hash functions are one order of magnitude faster than a classical set of hash functions.

**Figure 3:**
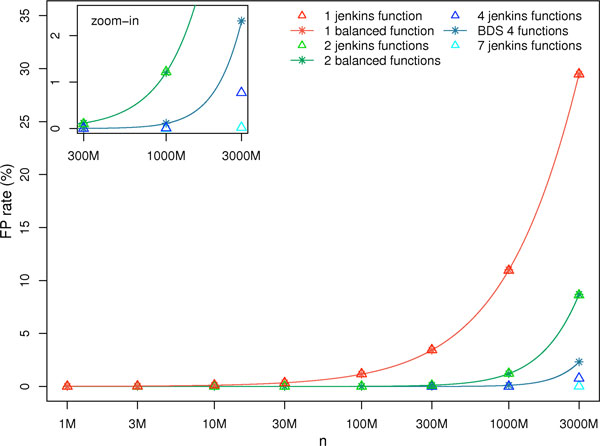
**Jenkins versus BDS false positive rate**. Comparison of FP rates between classical hash functions and the functions we used in the BDS. FP rate is plotted as a function of the number of indexed *k*-mers (in log scale), with *k *= 33. Plain lines correspond to theoretical predictions for the balanced functions (BDS), whereas star points and triangles correspond to empirical values obtained with simulations using respectively BDS functions and classical hash functions. The combination entitled "BDS 4 functions" is the one chosen for Compareads and is composed of the 3 balanced functions plus one unbalanced.

**FP rate comparison with other hash functions **We can see in Figure 3 that the FP rate of classical hash functions follows the FP rate of our balanced functions (it follows the equation 5 with the exponent 3 being replaced by the number of functions used). However it diverges with more than 3 functions, as we could not add other balanced functions and we added in place unbalanced ones which have higher FP rates. Even if, for more than three functions, classical hash functions produce less FP, the difference with our BDS structure is small: for 1 billion indexed *k*-mers, combinations of 4 classical functions give 0.01% FP on average, compared to 0.114%. We chose to have a slithly higher FP rate, but with a significant gain in computing time.

**Comparison with a classical Bloom filter **A classical Bloom filter requires a fixed amount of memory to index *n k*-mers. Evaluating the Bloom filter memory using formula from Section "*Bloom filter*" for one billion elements, with a false positive rate of 0.114%, yields 1.8 GB of memory. While this is twice smaller than the BDS, a classical Bloom filter would require classical hash functions. However, as shown above, classical hash functions are an order of magnitude slower to compute.

### Comparison with a classical approach using BLAST

Our approach is an heuristic based on shared *k*-mers between reads. Here we compare Compareads with a well-established method, BLAST[21], that is based on sequence alignment. The dataset used is composed of 15 bacterial metagenomes obtained from fresh water with three different conditions of Carbon/Nitrogen ratio (unpublished data). On average, each sample is composed of 176409 reads with an average of 400 nucleotides per read (Roche 454 technology).

Both BLAST and Compareads were used to compute all of the 120 pairwise intersections between the 15 datasets. BLAST was configured to find similar sequences between two samples with a local alignment greater than 80 nucleotides and more than 90% of sequence identity. Compareads was used to find sequences sharing respectively *t *= 1, 4 and 10 *k*-mers of 33 nucleotides. As shown in Table 1, computing one intersection between two samples using Compareads is more than 30 times faster than using BLAST for a close total number of similar reads.

**Table 1:** Comparison between Compareads and BLAST.

	Total Time (min)	Mean Time for one intersection (s)	Reads Found
BLAST	7200	3600	33 400 091

Compareads 1 ∗ 33	238	119	35 898 023

Compareads 4 ∗ 33	230	115	31 997 243

Compareads 10 ∗ 33	228	114	21 350 268

CPU time per intersection and global CPU time using a single core of an Intel^® ^Xeon^® ^CPU X5550 at 2.67GHz. **Reads Found **corresponds to the total number of similar reads in all the 120 intersections.

**Figure 4:**
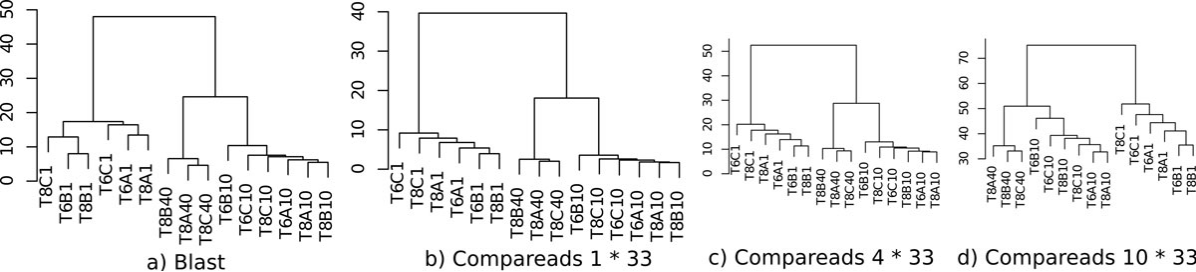
**Clustering based on Compareads and BLAST results**. Representation of hierarchical clustering based on pairwise intersections between all samples using BLAST (a) and Compareads (b, c, d).

For each experiment, samples were hierarchically clustered based on their pairwise similarity scores and then drawn as a dendrogram. As shown in Figure 4, the dendrogram obtained with the BLAST approach **(a) **is slightly different but the three main branches are the same than with the Compareads approach **(b)**. Interestingly, these branches discriminate three groups of samples corresponding to the three different biological conditions indicated by 1, 10 and 40 in the samples names: 1 corresponds to addition of Carbon in the water, 10 stands for normal condition and 40 for introduction of Nitrogen. Notably, all dendrograms based on Compareads approach **(b, c, d) **show a similar organization. Increasing the number of shared *k*-mers leads to be more stringent and decreases the number of similar reads but do not affect the global organization of the dendrogram, demonstrating the robustness of our similarity measure.

### Applying Compareads to Global Ocean metagenomic samples

**Figure 5:**
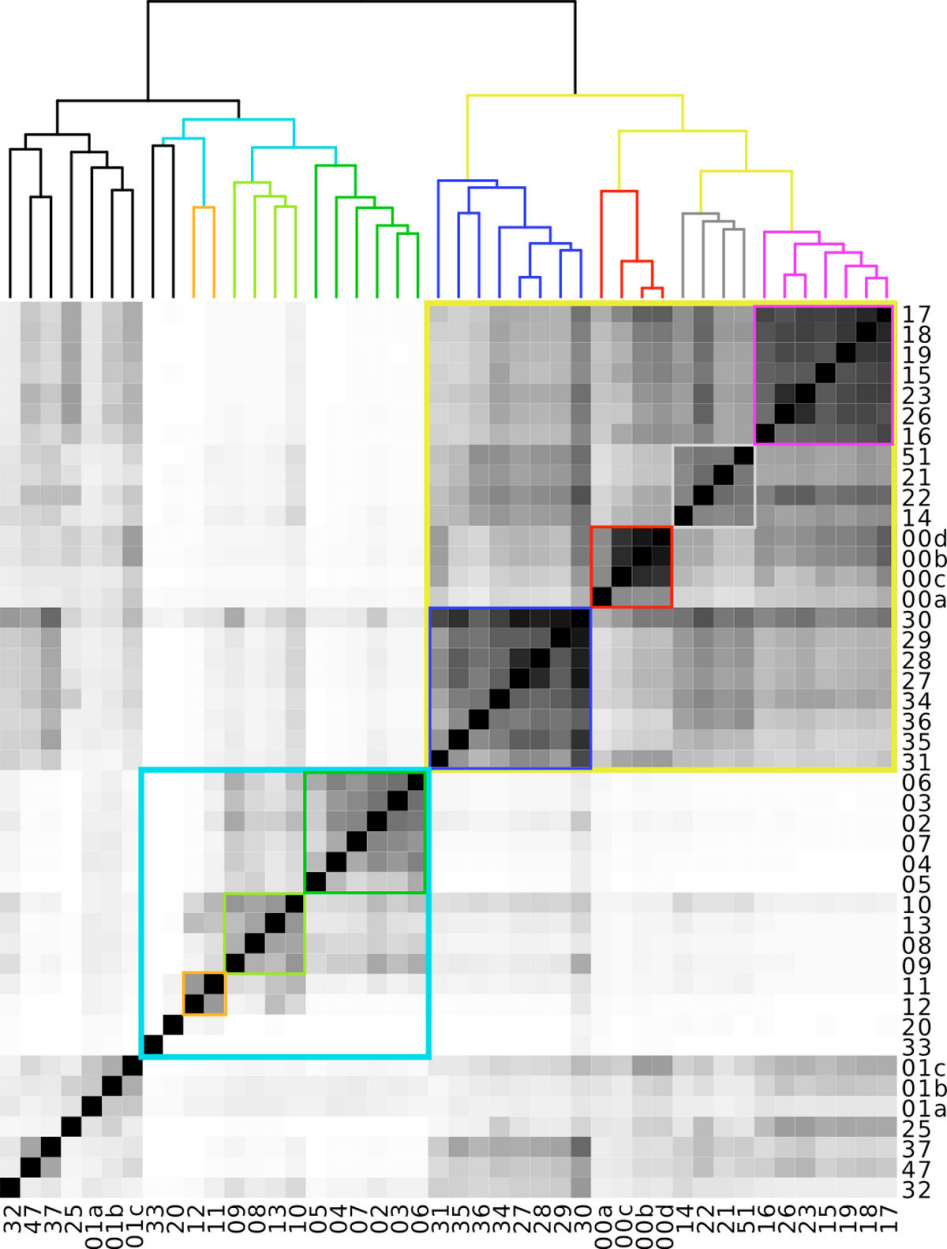
**Heatmap of intersections in Global Ocean Sampling**. Similarity matrix resulting from the comparison of 44 samples from The *Sorcerer II *Global Ocean Sampling Expedition using Compareads. Grey levels correspond to similarity levels, intersections with more than 50% of similarity are in black. The two main groups, in turquoise-blue and yellow, correspond respectively to north American east coast and tropical samples.

We tested Compareads on a larger and famous public dataset from the Global Ocean Sampling (The Sorcerer II expedition) [22]. It is composed of 44 samples from the microbial world of seawater, collected across several thousand of kilometers from the Northwest Atlantic through the Eastern Tropical Pacific oceans and for which an analysis of similarity between samples has been done [22]. The whole dataset is composed of 44 samples containing each on average 174759 long reads (1249 nucleotides per read on average, Sanger technology). Compareads computed all of the 990 intersections in 72 hours and half: on average, one intersection was performed in 4 minutes and 23 seconds on a single core of an Intel^® ^Xeon^® ^CPU X5550 at 2.67GHz. Results presented in Figure 5 are highly similar to those presented in the original publication [22], p.418. Two main groups are well discriminated. The first one, represented in turquoise-blue, groups together almost all samples coming from temperate seawater of the North American East Coast except the 14 one, consistently with the original study. This group also contains two samples really different from all others: the first contains freshwater and the second hypersaline water. The dark-green part corresponds to samples coming from the north part while light-green one gathers samples from the south part. Orange samples correspond to estuary. All of those three groups are identical to the original study. The second main part, colored in yellow, groups together datasets of tropical and Sargasso seawater. The dark-blue part aggregates samples coming only from Galapagos Islands. Red square delimitates Sargasso Sea samples. On the original study, the sample 00a is not in this group. According to metadata, the gray part, like in the original publication, is composed of various samples. Finally, purple samples regroup both Caribbean Sea and some Open Ocean datasets, as the original study.

Those results show that Compareads can also be used on Sanger reads and deliver reliable biological conclusions. Indeed, despite of false positives and the simple definition of similarity, we were able to retrieve the classification of metagenomes according to their geographical origin.

## Conclusion

Motivated by *de novo *comparative metagenomics, this paper proposes two main contributions. The first one is a data structure based on Bloom filters that can index, for instance up to one billion distinct words of length 33 (33-mers) using 4Gb of memory, with an error rate of 0.11%, and that is faster to build and request, to the best of our knowledge, that any other existing data structure. The second main contribution is a software, called Compareads which uses this data structure to efficiently perform *de novo *intensive comparisons of huge metagenomic datasets generated by High Throughput Sequencers. We have shown that this approach enables to retrieve and classify differences in species content between metagenomic samples. For this kind of comparison, our approach is much faster than alternative ones such as BLAST and thus enables to scale to huge datasets. We furthermore tested the scalability of Compareads on a large oceanic dataset (unpublished), from the Tara Ocean expedition [15]; it is composed of 31 metagenomes and contains overall 3.5 billions of Illumina short reads (108bp). Each intersection was performed in 10 hours and 55 minutes in average using 4Gb of memory. Such features enabled us to compute the $\frac{31*32}{2}=496$ metagenome datasets intersections in 6 days and 10 hours using 50 cores of Intel^® ^Xeon^® ^CPU X5550 at 2.67GHz. This would have been unfeasible with any other known existing tools (based on results Section "*Comparison with a classical approach using *BLAST", BLAST is about 30 times longer and would take more than 6 months to complete this task with the same resources).

Compareads has been conceived for being parallelizable both at fine and coarse grained levels. Future work will consist in implementing a parallel version exploiting multi-core and GPU chips. Compareads is released under the CeCILL license and can be freely downloaded from http://alcovna.genouest.org/compareads/.

## Competing interests

The authors declare that they have no competing interests.

## Author's contributions

DL and PP initiated the work. RC and CL provided expertise about Bloom filters datastructures and their statistical aspects. NM and PP made the implementations. NM, CL, DL and PP performed the experiments. All authors participated to the redaction and approved the final manuscript.

## Supplementary Material

Additional file 1 **Theoretical details for the false positive rate**. Details about how theoretical false positive results were obtained. Theoretical details for the false positive rate. Details about how theoretical false positive results were obtained. Click here for file

Additional file 2 **Empirical estimation of false positive rate**. Details about how empirical false positive results were obtained. Empirical estimation of false positive rate. Details about how empirical false positive results were obtained. Click here for file
