# Supplementary material for: Compareads: comparing huge metagenomic experiments
Source: BMC Bioinformatics. 2012 Dec 19;13(Suppl 19):S10. doi: 10.1186/1471-2105-13-S19-S10 (PMC3526429; doi:10.1186/1471-2105-13-S19-S10)
Supplement: Additional file 1 — Theoretical details for the false positive rate. Details about how theoretical false positive results were obtained. Theoretical details for the false positive rate. Details about how theoretical false positive results were obtained. [file 1471-2105-13-S19-S10-S1.PDF]

Additional file 1 — Theoretical details for the false positive rate

As exposed in Section "*The Bloom Data Structure index*", the BDS index is a probabilistic data structure, that may consider a k-mer as indexed while this is not the case (*i.e.* a false positive). Here, we tried to express the false positive rate for each hash function that we defined in Section "*Particular hash functions*" and their combinations with respect to the parameter k and the number n of distinct indexed k-mers.

False positive probablity for each function Assuming the base composition of the indexed and query k-mers is unbiaised, we can easily compute the probability, $P_{FP}(f,k,n)$, for any query k-mer to be a false positive with one of the seven hash functions, f. This probability depends on the number of distinct k-mers sharing the same hash code. We can notice that for the balanced functions, f_1, f_2 and f_3, each 0 and 1 value can come from exactly 2 distinct nucleotides, thus the number of k-mers sharing the same hash code is the same for all k-mers and equals: 2^k. The probability for 2 k-mers to have distinct hash codes is then $1 - \frac{2^k}{4^k} = 1 - \frac{1}{2^k}$, and therefore the probability to have at least one k-mer among the n that are indexed sharing the same hash code is:

$$\forall i \in \{1,2,3\} P_{FP}(f_i,k,n) = 1 - (1 - \frac{1}{2^k})^n \qquad ((3))$$

Note that this corresponds to the false positive probability of any hash function that distributes the hash codes uniformly in a 2^k bit-array, such as those inspired of Jenkins functions, used as a comparison in Section "*Comparison with other hash functions and with a classical Bloom filter*".

As for the unbalanced functions, since the 0 bit-value encodes only one base, the number of k-mers sharing the same hash code depends on the number of 0 in the hash code of the query. For a given query k-mer with a hash code having x 0 the above probability for functions f_4, f_5, f_6 and f_7 becomes: $1-(1-(\frac{1}{4})^x(\frac{3}{4})^{(k-x)})^n$. To obtain the probability for any kmer, we have to sum over the different values of x the latter probability weigthed by the probablity for a k-mer hash code to have x 0. The composition of a given base in a k-mer of length k, assuming unbiased nucleotide composition, follows a binomial distribution, thus we get:

$$\forall i \in \{4,5,6,7\} \quad P_{FP}(f_i,k,n) = \sum_{x=0}^{k} \binom{k}{x} a_x (1-(1-a_x)^n) \qquad with \quad a_x = (\frac{1}{4})^x(\frac{3}{4})^{k-x} \qquad ((4))$$

$\binom{k}{x}$ being the binomial coefficient, ie $\binom{k}{x} = \frac{k!}{x!(k-x)!}$.

We can see in Figure 2 that balanced functions give much less false positives than unbalanced ones. This can be explained by the fact that for unbalanced functions, for a given k-mer with a "normal" composition and thus 25% of 0 in its hash-code, there are many more k-mers with the same hash-code than for a balanced

function: $3^{\frac{3k}{4}} \gg 2^k$.

FP probablity for a combination of functions When combining several functions in our BDS, in order to have a false positive for a query k-mer all functions must return a false positive. As concerns the balanced function, we can easily see that for a given kmer, we can not find another k-mer that is a false positive simultaneously for any two 2 of these functions. In other words, there do not exist two distinct k-mers that have the same couple of hash codes with any two of these functions. This implies that the probability of having a false positive with one function does not depend on the result with another function, apart from the fact that the effective number of indexed k-mers that can be a false positive (n in equation 3) is reduced: indeed if x k-mers have the same hash code for one function, these k-mers have a null probability of having the same hash code for another function. Note that this effect can be neglicted given that n is very large. Therefore the product of individual probabilities for each balanced function gives the following upper bound:

$$P_{FP}(f_1 \cap f_2 \cap f_3, k, n) \lesssim (1 - (1 - \frac{1}{2^k})^n)^3 \qquad ((5))$$

Concerning the unbalanced functions, this independence property is lost, since it is possible to find a single k-mer that is a false positive for at least 2 of the unbalanced functions, or for one balanced function and at least one unbalanced. Therefore we could not figure out the theoretical false positive rate, or even an upper bound.
