# Supplementary material for: Compareads: comparing huge metagenomic experiments
Source: BMC Bioinformatics. 2012 Dec 19;13(Suppl 19):S10. doi: 10.1186/1471-2105-13-S19-S10 (PMC3526429; doi:10.1186/1471-2105-13-S19-S10)
Supplement: Additional file 2 — Empirical estimation of false positive rate. Details about how empirical false positive results were obtained. Empirical estimation of false positive rate. Details about how empirical false positive results were obtained. [file 1471-2105-13-S19-S10-S2.PDF]

Additional file 2 — Empirical estimation of false positive rate

For estimating the false positive rate of the BDS filled up with n k-mers, we made the following experiment. We generated n+100000 distinct random k-mers, used first for filling up the BDS with n k-mers and then for querying the BDS with the 100000 remaining k-mers. All k-mers being distinct, if the BDS answers "yes" while querying the presence of a k-mer, it is a false positive.

The generation of a huge set of x distinct random k-mers (with $x < 4^k$) is not trivial. This was done by dividing the space of 4^k k-mers into x non overlapping blocks, and them by picking up a random k-mer into each block. This method enables to uniformly cover the whole k-mer space.
